# Tanshinone IIA Inhibits Glutamate-Induced Oxidative Toxicity through Prevention of Mitochondrial Dysfunction and Suppression of MAPK Activation in SH-SY5Y Human Neuroblastoma Cells

**DOI:** 10.1155/2017/4517486

**Published:** 2017-06-11

**Authors:** Haifeng Li, Wenjing Han, Hongyu Wang, Fei Ding, Lingyun Xiao, Ruona Shi, Liping Ai, Zebo Huang

**Affiliations:** ^1^Center for Bioresources & Drug Discovery and School of Biosciences & Biopharmaceutics, Guangdong Pharmaceutical University, Guangzhou 510006, China; ^2^School of Pharmaceutical Sciences, Wuhan University, Wuhan 430071, China; ^3^Hunan Auragene Biotech Co., Ltd., Changsha 410013, China; ^4^Guangdong Province Key Laboratory for Biotechnology Drug Candidates, Guangdong Pharmaceutical University, Guangzhou 510006, China

## Abstract

Glutamate excitotoxicity is associated with many neurological diseases, including cerebral ischemia and neurodegenerative diseases. Tanshinone IIA, a diterpenoid naphthoquinone from *Salvia miltiorrhiza*, has been shown to suppress presynaptic glutamate release, but its protective mechanism against glutamate-induced neurotoxicity is lacking. Using SH-SY5Y human neuroblastoma cells, we show here that excessive glutamate exposure decreases cell viability and proliferation and increases LDH release. Pretreatment with tanshinone IIA, however, prevents the decrease in cell viability and proliferation and the increase in LDH release induced by glutamate. Tanshinone IIA also attenuates glutamate-induced oxidative stress by reducing reactive oxygen species level and malondialdehyde and protein carbonyl contents and by enhancing activities and protein levels of superoxide dismutase and catalase. We then show that tanshinone IIA prevents glutamate-induced mitochondrial dysfunction by increasing mitochondrial membrane potential and ATP content and by reducing mitochondrial protein carbonyl content. Moreover, tanshinone IIA can inhibit glutamate-induced apoptosis through regulation of apoptosis-related protein expression and MAPK activation, including elevation of Bcl-2 protein level, decrease in Bax and cleaved caspase-3 levels, and suppression of JNK and p38 MAPK activation. Collectively, our findings demonstrate that tanshinone IIA protects SH-SY5Y cells against glutamate toxicity by reducing oxidative stress and regulating apoptosis and MAPK pathways.

## 1. Introduction

As the global population ages, increasing prevalence of age-related neurological disorders, such as Alzheimer's disease and cerebral ischemia, has become a major health concern worldwide [1, 2]. Over the past few decades, studies have uncovered that several free amino acids, including glutamate, aspartate, and cysteine, act as excitatory neurotransmitters in the mammalian cerebral cortex and hippocampus [3]. The excitatory amino acid system is critical for a number of important neurological functions involving learning, memory, and higher cognitive processes [4], and its malfunction may lead to the onset and progression of many neurological diseases [2, 4–6]. For example, glutamate, the major excitatory neurotransmitter in the brain and spinal cord, plays a pivotal role in basal excitatory synaptic transmission and plasticity [5]. Under normal conditions, an excitatory response is generated via controlled synaptic release of glutamate followed by an interaction of glutamate with its receptors. However, excessive glutamate caused by overrelease or prolonged exposure can overstimulate glutamate receptors and thus induce calcium overload, resulting in neuronal injury and death as demonstrated in both experimental models and humans [7]. The neurotoxicity induced by excessive glutamate, referred to as excitotoxicity, has been implicated in several neurodegenerative disorders [6].

A number of studies have demonstrated that the molecular mechanisms underlying glutamate neurotoxicity primarily involve oxidative stress and mitochondrial dysfunction [8, 9]. Oxidative stress is essentially caused by an imbalance between free radical formation and elimination, that is, increased reactive oxygen species (ROS) production and/or reduced antioxidant defense. Persistent exogenous insults such as glutamate exposure can trigger an increase in mitochondrial membrane permeability through calcium influx and further promote ROS generation, making cellular lipids, proteins, and DNA more vulnerable to oxidative damage [9, 10]. Mitochondrial dysfunction also causes a decrease in energy production and promotes release of proapoptotic cytochrome c into the cytoplasm, leading to activation of cell death pathways [11]. In fact, a cascade of signaling events such as mitogen-activated protein kinases (MAPK) is shown to play an important role in glutamate-mediated neuronal death [12–14]. For example, it has been shown that activation of MAPK is involved in glutamate toxicity to dopaminergic neurons in rat mesencephalic cultures, and specific MAPK inhibitors are able to prevent glutamate-induced cytotoxicity and apoptosis [13, 14]. Therefore, reducing oxidative stress and manipulating excitotoxicity-related signaling pathways are promising strategies to alleviate glutamate neurotoxicity.

Tanshinone IIA is a diterpene quinone isolated from the roots of *Salvia miltiorrhiza*, which is a well-known Chinese medicine for promoting blood circulation and relieving vessel stasis [15]. The sulfonic sodium of tanshinone IIA is widely used to treat cardiovascular and cerebrovascular diseases [16]. Moreover, recent studies have shown that tanshinone IIA has beneficial effects on animal models of neurodegenerative disorders [17, 18]. For example, tanshinone IIA is capable of attenuating memory impairment in amyloid precursor protein/presenilin 1 transgenic mice via reducing accumulation of amyloid *β*-protein, a critical pathogenic protein in Alzheimer's disease, and activating synthesis of a synaptic brain-derived neurotrophic factor [18]. Interestingly, tanshinone IIA is recently found to suppress glutamate release from rat cortical synaptosomes by inhibiting presynaptic voltage-dependent calcium entry [19], demonstrating its ability to regulate the excitatory amino acid system. However, the effect of tanshinone IIA on glutamate-induced neurotoxicity and the underlying mechanisms are lacking. In this study, we investigated the protective effect of tanshinone IIA against glutamate-mediated toxicity using human neuroblastoma cell line SH-SY5Y and attempted to explore its neuroprotective mechanisms, including reduction of oxidative stress and regulation of signaling pathways related to neuronal apoptosis.

## 2. Materials and Methods

### 2.1. Cell Culture

Human neuroblastoma cell line SH-SY5Y was obtained from American Type Culture Collection (ATCC) and cultured in DMEM/F12 medium supplemented with 10% fetal bovine serum, 100 U/mL penicillin, 100 *μ*g/mL streptomycin, and 2 mM *L*-glutamate (Sigma, St. Louis, MO, USA) at 37°C in a humidified atmosphere of 5% CO_2_. The culture medium was replaced with fresh medium every other day, and the cells were passaged by trypsinization when the confluence reached about 80%.

### 2.2. Determination of Cell Viability

The viability of SH-SY5Y cells was measured by a 3-(4,5-dimethylthiazol-2-yl)-2,5-diphenyl tetrazolium bromide (MTT) reduction assay [20] and trypan blue exclusion assay [21] as previously described. For the MTT assay, 100 *μ*L of cells was seeded at a density of 1 × 10^5^ cells/mL in 96-well plates, incubated at 37°C for 24 h, and then treated with tanshinone IIA (Sigma, St. Louis, MO, USA) at the indicated concentrations (2.5–10.0 *μ*M) for 24 h. After the addition of 10 *μ*L *L*-glutamate (final concentration, 10 mM), the cells were further incubated for 24 h. Then, 10 *μ*L of 5 mg/mL MTT was added to each well and the plates were incubated at 37°C for 3 h. The medium was then removed, and the formazan crystals were dissolved with 150 *μ*L of DMSO. The absorbance at 570 nm was measured by a microplate reader (Thermo Fisher, Waltham, MA, USA). For the trypan blue exclusion assay, 2 mL of cells was seeded at a density of 1 × 10^5^ cells/mL in 6-well plates and incubated at 37°C for 24 h. After treatment with tanshinone IIA and glutamate as described above, the cells were collected by trypsinization and centrifugation and resuspended in phosphate-buffered saline (PBS; 50 mM, pH 7.8). The cell suspension was then mixed with 0.4% trypan blue solution and incubated at room temperature for 3 min. Immediately after the staining, trypan blue-positive and trypan blue-negative cells were counted using a hemocytometer under a microscope. Approximately 500 cells were counted for each group, and the trypan blue exclusion rate was calculated as a percentage of trypan blue-negative cells relative to the total cells. Data were normalized to the cells without tanshinone IIA treatment and glutamate exposure.

### 2.3. Determination of Cell Proliferation

The proliferation of SH-SY5Y cells was measured using a 5-bromo-2′-deoxyuridine (BrdU) incorporation assay kit (KeyGEN, Nanjing, China) as previously described [22]. Briefly, the cells were grown and treated with tanshinone IIA and glutamate in 6-well plates as described in the trypan blue exclusion assay. Then, BrdU solution was added to each well at a final concentration of 10 *μ*M and incubated further at 37°C for 12 h. After removing the culture medium, the cells were fixed and the DNA was denatured according to the instructions of the assay kit. The cells were then incubated with allophycocyanin-conjugated anti-BrdU antibody at 4°C for 30 min in the dark. BrdU-incorporated cells were counted by a fluorescence microscope (Olympus, Tokyo, Japan) from ~500 total cells, and the BrdU incorporation rate was calculated as a percentage of BrdU-incorporated cells relative to the total cells. The data were normalized to the cells without tanshinone IIA treatment and glutamate exposure.

### 2.4. Measurement of Lactate Dehydrogenase Release

The level of lactate dehydrogenase (LDH) released from damaged cells into medium was measured as previously described [23]. Briefly, SH-SY5Y cells were treated with tanshinone IIA and glutamate as described above, and the cell-free culture supernatants were collected. After incubation at room temperature for 30 min in the dark with the agents provided by the LDH assay kit (Beyotime, Shanghai, China) according to its protocol, the culture supernatants were subjected to measurement of LDH activity using the microplate reader at 490 nm. The relative level of LDH release was normalized to the activity of LDH in the culture supernatant of cells without tanshinone IIA treatment and glutamate exposure.

### 2.5. Determination of ROS Level

The cellular ROS level was measured using a 2′,7′-dichlorofluorescin diacetate (DCFH-DA) fluorescent probe as described [24]. Briefly, SH-SY5Y cells were treated with 2.5–10.0 *μ*M tanshinone IIA followed by 10 mM glutamate exposure as described above in black 96-well plates. The cells were then washed with ice-cold PBS and resuspended in 90 *μ*L of PBS. After adding 10 *μ*L of 10 *μ*M DCFH-DA, the cells were incubated at 37°C for 15 min in the dark. The 2′,7′-dichlorofluorescin (DCF) fluorescence was then measured using a Fluoroskan Ascent FL microplate reader (Thermo, Waltham, MA, USA) with an excitation of 485 nm and an emission of 535 nm.

### 2.6. Measurement of Malondialdehyde and Protein Carbonyl Contents, Antioxidant Enzyme Activities, and Antioxidant Enzyme Levels

The contents of the lipid peroxidation product malondialdehyde (MDA) [25] and the protein carbonyl groups [26] as well as the activities of superoxide dismutase (SOD) [25] and catalase (CAT) [25] and the protein levels of SOD [27] and CAT [28] were determined as described previously. Briefly, the cells were treated with 2.5–10.0 *μ*M tanshinone IIA as described above in 6-well plates prior to 10 mM glutamate exposure and then harvested and lysed in 0.5 mL of lysis buffer (0.5% Triton X-100 in PBS, pH 7.0) with sonication on ice. The homogenate was centrifuged at 12,000*g* for 10 min at 4°C, and the supernatant was collected to determine protein carbonyl content (Jiancheng, Nanjing, China), MDA content, SOD and CAT activities (Beyotime, Shanghai, China), SOD protein level (Cloud-Clone, Houston, TX, USA), and CAT protein level (Cusabio, Wuhan, China) using assay kits, respectively. For determination of mitochondrial protein carbonyl content, the mitochondria were first isolated from SH-SY5Y cells and then lysed in the lysis buffer to obtain the supernatant according to the instructions of the mitochondria isolation kit (Beyotime, Jiangsu, China) and the protein carbonyl assay kit. Protein content of the supernatants was determined using the BCA protein assay kit (Thermo Fisher, Waltham, MA, USA). The protein carbonyl and MDA contents were expressed as pmol/mg proteins and nmol/mg proteins, respectively, and the antioxidant enzyme activities and levels were expressed as U/mg proteins and ng/mg proteins, respectively.

### 2.7. Determination of Mitochondrial Membrane Potential

The fluorescent probe JC-1 exists as a green fluorescent monomer in cells at low mitochondrial membrane potential (MMP) and forms red fluorescent aggregates at high MMP and thus was used to measure MMP as described [29]. The SH-SY5Y cells were treated with tanshinone IIA prior to glutamate exposure in 96-well plates as described above. The culture medium was then removed, and the cells were further incubated with 50 *μ*L of Hank's solution containing 10 mg/mL JC-1 for 30 min at 37°C. After removal of the Hank's solution, the cells were washed and resuspended in 100 *μ*L of PBS. The green fluorescence of JC-1 monomer (excitation at 490 nm, emission at 530 nm) and the red fluorescence of JC-1 oligomer (excitation at 525 nm, emission at 590 nm) were measured using the Fluoroskan Ascent FL microplate reader. The data were presented as a red/green fluorescence ratio.

### 2.8. Measurement of ATP Content

ATP content was measured using an ATP assay kit (Beyotime, Shanghai, China) as described [30]. After tanshinone IIA pretreatment and glutamate exposure as described above, the SH-SY5Y cells were harvested from 6-well plates and lysed using 100 *μ*L of ATP-releasing reagent provided in the assay kit. The homogenate was centrifuged at 12,000*g* for 10 min at 4°C, and 20 *μ*L aliquots of the supernatant was transferred to white 96-well plates. Then, 100 *μ*L of ATP detection solution was added to each well, and the chemiluminescence was monitored using the Fluoroskan Ascent FL microplate reader. A calibration curve made with ATP standards between 0.01 *μ*M and 10 *μ*M was used to calculate ATP content, which was normalized by protein content.

### 2.9. Flow Cytometry Analysis of Cell Apoptosis

Apoptotic cells were quantified using Annexin V/propidium iodide (PI) staining as described previously [23]. Briefly, 3 mL of SH-SY5Y cells was seeded at a density of 1 × 10^5^ cells/mL in 6-well plates for 24 h and then treated with 2.5–10.0 *μ*M tanshinone IIA followed by 10 mM glutamate exposure as described above. After treatment, the cells were harvested and resuspended in PBS, and then, 5 *μ*L of 20 *μ*g/mL Annexin V-FITC was added into 195 *μ*L of cell suspension. The samples were mixed gently and incubated for 10 min at room temperature in the dark. Subsequently, 10 *μ*L of 20 *μ*g/mL PI was added, and each sample was incubated further for 10 min. The samples were then immediately analyzed using a flow cytometer (Becton-Dickinson, Rutherford, NJ, USA) with an excitation of 488 nm and an emission of 530 nm. At least 10,000 cells within the gated region were analyzed. The apoptosis rate was calculated as the percentage of Annexin V-positive cells divided by the total cells in the gated region.

### 2.10. Western Blot Analysis

Western blot analysis was performed as described previously [31]. After pretreatment with 2.5–10.0 *μ*M tanshinone IIA and exposure to 10 mM glutamate, the SH-SY5Y cells were harvested from 6-well plates and lysed on ice. The homogenate was centrifuged at 4°C, and the supernatant was collected. Protein concentration was quantified using the BCA protein assay kit as described above. Equal amount of proteins from each sample was subjected to 12% SDS-PAGE and then transferred to PVDF membranes. Nonspecific binding was blocked with 10% skim milk in Tris-buffered saline containing 0.2% (*v*/*v*) Tween-20 (TBS-T) for 1 h. The blots were incubated with the primary antibodies against Bax (Abcam, Cambridge, UK), Bcl-2 (Abcam), caspase-3 (Abcam), total p38 MAPK (Abcam), phospho-p38 MAPK (Abcam), total JNK1/2 (Cell Signaling Technology, Danvers, MA, USA), phospho-JNK1/2 (Cell Signaling Technology), total ERK1/2 (Abclonal, Cambridge, MA, USA), and phospho-ERK1/2 (Abclonal) overnight at 4°C. After incubation, the blots were washed with TBS-T and further incubated with horseradish peroxidase-labeled anti-rabbit IgG (Cell Signaling Technology) for 1 h at room temperature. After washing with TBS-T, the protein bands were visualized using the ECL Western detection reagents (Millipore, Billerica, MA, USA). The monoclonal antibody *β*-actin (Cell Signaling Technology) was used as a reference. The images were obtained with an Epson V300 scanner (Palo Alto, CA, USA), and the blot densitometry was performed using ImageJ software (National Institutes of Health, Bethesda, MD, USA).

### 2.11. Statistical Analysis

The statistical analysis was performed by using GraphPad Prism 5.01 for Windows (GraphPad Software, San Diego, CA, USA). Data were analyzed by one-way analysis of variance (ANOVA) and Dunnett's post hoc test. A probability value of *p* < 0.05 was considered to be statistically significant. All experiments were performed at least three times.

## 3. Results

### 3.1. Tanshinone IIA Protects SH-SY5Y Neuroblastoma Cells against Glutamate Toxicity

**Figure 1:**
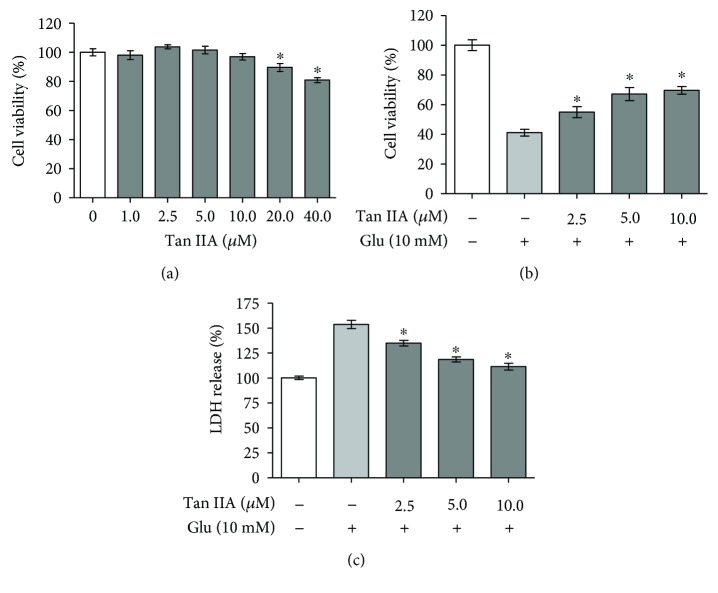
Effect of tanshinone IIA on glutamate cytotoxicity in SH-SY5Y cells. (a) Relative viability of SH-SY5Y cells treated with tanshinone IIA at the indicated concentrations at 37°C for 24 h. (b) Relative viability of SH-SY5Y cells pretreated with tanshinone IIA at the indicated concentrations for 24 h and then exposed to 10 mM glutamate for another 24 h. (c) Relative level of LDH release of the SH-SY5Y cells treated as in (b). All data are normalized to the cells without tanshinone IIA treatment and glutamate exposure and presented as mean ± SEM of three independent experiments. Tan IIA: tanshinone IIA; Glu: glutamate. ^∗^*p* < 0.05 compared to the cells without tanshinone IIA treatment (a) and the cells exposed to glutamate alone ((b) and (c)).

To evaluate the protective effect of tanshinone IIA on glutamate-exposed SH-SY5Y neuroblastoma cells, we examined the cell viability using the MTT colorimetric assay. Tanshinone IIA was first applied alone to SH-SY5Y cells to determine its concentration range to be used in the cells. As shown in Figure 1(a), the cell viability was noticeably reduced after treatment for 24 h with tanshinone IIA at ≥20 *μ*M while that of the cells treated with 1–10 *μ*M of tanshinone IIA remained almost unchanged as compared to that of the control cells. Therefore, tanshinone IIA was used at <20 *μ*M in the following experiments. To assess the effect of tanshinone IIA on glutamate toxicity, the SH-SY5Y cells were first pretreated with tanshinone IIA at the indicated concentrations as described above and then exposed to 10 mM glutamate for 24 h. As shown in Figure 1(b), when the cells were exposed to glutamate without tanshinone IIA pretreatment, the cell viability was reduced to ~40% as compared to that of the unexposed cells. When the cells were pretreated with 2.5–10.0 *μ*M tanshinone IIA and then exposed to 10 mM glutamate, the cell viability was increased to >50% as compared to that of the cells exposed to glutamate alone (*p* < 0.05). As the cytotoxic action of glutamate is known to be associated with disruption of cell membrane integrity [32], we further investigated whether tanshinone IIA was able to reduce the release of intracellular LDH, an important indicator of membrane injury, in glutamate-exposed cells. When the SH-SY5Y cells were exposed to glutamate alone, the relative release of LDH was increased to ~150% as compared to that of the control (Figure 1(c)). Interestingly, the release of LDH in glutamate-exposed cells was significantly reduced when the cells were pretreated with tanshinone IIA at the indicated concentrations as described above, suggesting that tanshinone IIA is able to alleviate cell membrane damage induced by glutamate. In addition to MTT and LDH assays, which have demonstrated the protective effect of tanshinone IIA against glutamate-induced cytotoxicity by reducing disruption of membrane integrity, we also determined the viability of SH-SY5Y cells by directly counting viable cells under a microscope after trypan blue staining. As shown in Figure 1S(a) available online at https://doi.org/10.1155/2017/4517486, the reduction of trypan blue exclusion rate was inhibited by tanshinone IIA in glutamate-exposed cells, further demonstrating the protective activity of tanshinone IIA against glutamate toxicity. We also performed a BrdU incorporation assay to further investigate the effect of tanshinone IIA on cell proliferation under glutamate challenge and found that the BrdU incorporation rate was reduced in glutamate-exposed SH-SY5Y cells by pretreatment with tanshinone IIA (Figure 1S(b)), again indicating the protective effect of tanshinone IIA against glutamate cytotoxicity.

### 3.2. Tanshinone IIA Reduces Glutamate-Induced Accumulation of ROS, Malondialdehyde, and Carbonylated Proteins in SH-SY5Y Cells

As oxidative damage is shown to be implicated in glutamate-mediated neurotoxicity [8], we investigated whether the protective effect of tanshinone IIA against glutamate toxicity was associated with regulation of ROS level, a major cause of oxidative stress. The SH-SY5Y cells were treated with tanshinone IIA at the indicated concentrations for 24 h and then exposed to 10 mM glutamate for 24 h, and the ROS level was measured using a DCFH-DA fluorescent probe. As shown in Table 1, the intracellular ROS level, which is represented by DCF fluorescence intensity, was elevated after the cells were exposed to glutamate per se. Interestingly, the elevated ROS level was reduced in the glutamate-exposed cells by pretreatment with 2.5–10.0 *μ*M tanshinone IIA. To further explore the protective mechanism of tanshinone IIA against oxidative damage, we then examined its effect on the content of the lipid peroxidation product MDA, a well-known biomarker of ROS-mediated oxidative stress. As shown in Table 1, the MDA content of the glutamate-exposed cells was increased as compared to that of the control cells. When the cells were pretreated with 2.5–10.0 *μ*M tanshinone IIA, however, the increased MDA content was reduced. Moreover, we investigated the effect of tanshinone IIA on the content of protein carbonyl groups, a marker of oxidative modification of proteins, and found that the glutamate-induced increase in protein carbonyl content in SH-SY5Y cells was prevented by tanshinone IIA pretreatment (Table 1). Together, these results demonstrate that tanshinone IIA is capable of attenuating glutamate-mediated oxidative stress through reducing ROS level and inhibiting lipid and protein peroxidation.

### 3.3. Tanshinone IIA Increases Antioxidant Enzyme Activities and Levels in Glutamate-Exposed SH-SY5Y Cells

Endogenous antioxidant enzymes are known to play important roles in scavenging excessive ROS and maintenance of cellular redox balance [33]. Therefore, we tested whether the antioxidant effect of tanshinone IIA on glutamate-exposed SH-SY5Y cells was associated with the regulation of antioxidant enzymes such as SOD and CAT. As shown in Table 1, both SOD and CAT activities in the glutamate-exposed SH-SY5Y cells were decreased as compared to those in the control, but the decrease was reversed when the cells were pretreated with tanshinone IIA at the indicated concentrations. Using ELISA assays, we show that the protein levels of SOD and CAT were also reduced in SH-SY5Y cells exposed to glutamate alone as compared to the control, but the reduced SOD and CAT levels were restored after pretreatment with tanshinone IIA (Table 1). Together, these results suggest that regulation of the antioxidant enzymes, including both activity and expression, may contribute to the ROS scavenging effect of tanshinone IIA.

### 3.4. Tanshinone IIA Attenuates Mitochondrial Dysfunction in Glutamate-Exposed SH-SY5Y Cells

**Figure 2:**
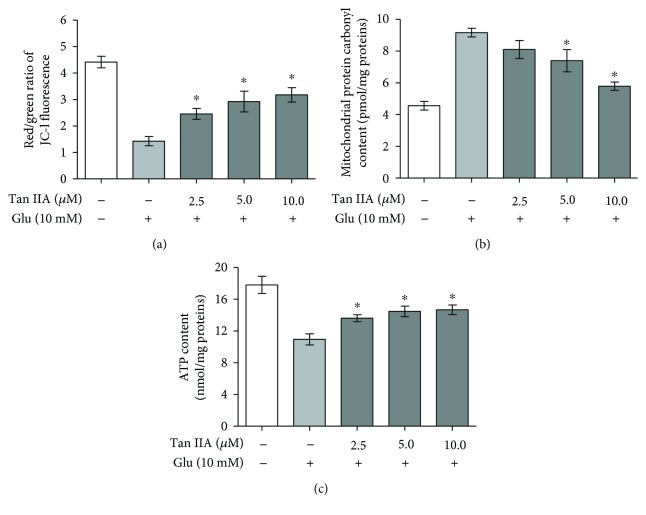
Effect of tanshinone IIA on mitochondrial membrane potential, protein carbonyl content, and ATP content in glutamate-exposed SH-SY5Y cells. The cells were pretreated with tanshinone IIA at the indicated concentrations and then exposed to 10 mM glutamate. (a) Mitochondrial membrane potential was determined using the JC-1 fluorescence probe and presented as the ratio of red to green fluorescence. (b) Mitochondrial protein carbonyl content was determined using a colorimetric assay. (c) Cellular ATP content was measured using a chemiluminescence method. The results are shown as mean ± SEM of three independent experiments. Tan IIA: tanshinone IIA; Glu: glutamate. ^∗^*p* < 0.05 compared to the cells exposed to glutamate alone.

As the mitochondria are sensitive targets of glutamate insult and oxidative damage [9, 11], we investigated the effect of tanshinone IIA on mitochondrial function by examining MMP, a marker of mitochondrial injury, with the JC-1 fluorescent probe. As shown in Figure 2(a), after the SH-SY5Y cells were exposed to 10 mM glutamate for 24 h, the red to green fluorescence ratio, which serves as an indicator of MMP, was markedly reduced as compared to that of the control. When the cells were pretreated with 2.5–10.0 *μ*M tanshinone IIA and then exposed to 10 mM glutamate as described above, the red to green fluorescence ratio was significantly increased as compared to that of the glutamate-exposed cells (*p* < 0.05), suggesting that tanshinone IIA is capable of alleviating glutamate-induced mitochondrial membrane damage. To further confirm this, we examined the effect of tanshinone IIA on the content of mitochondrial protein carbonyl groups, an indicator of mitochondrial protein damage. As shown in Figure 2(b), the mitochondrial protein carbonyl content was increased after the SH-SY5Y cells were exposed to glutamate, but the increase was reduced by pretreatment with tanshinone IIA, indicating that tanshinone IIA can inhibit oxidative modification of mitochondrial proteins induced by glutamate. Since mitochondrial dysfunction may lead to a defect of ATP synthesis, we further examined the effect of tanshinone IIA on ATP content under glutamate intoxication. As shown in Figure 2(c), the ATP content was reduced in SH-SY5Y cells treated with 10 mM glutamate as compared to the control cells, but the ATP content of the glutamate-exposed cells was increased after pretreatment with 2.5–10.0 *μ*M tanshinone IIA. Together, these results indicate that tanshinone IIA can maintain mitochondrial function under glutamate insult.

### 3.5. Tanshinone IIA Inhibits Glutamate-Induced Apoptosis in SH-SY5Y Cells

**Figure 3:**
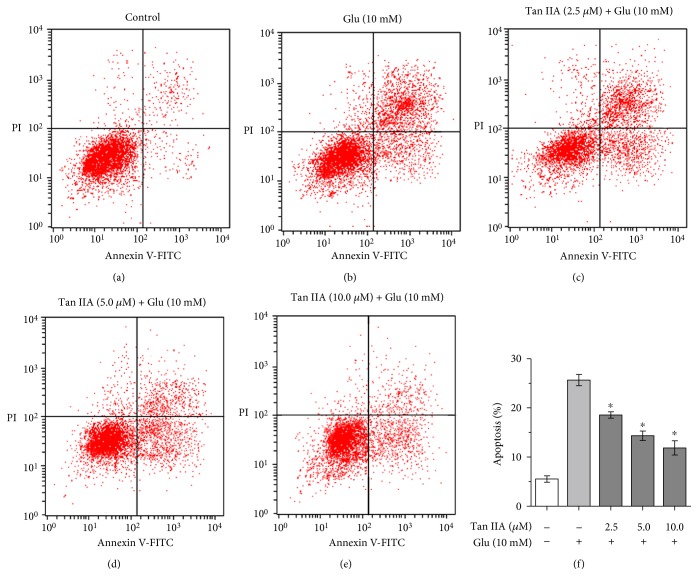
Effect of tanshinone IIA on glutamate-induced apoptosis in SH-SY5Y cells. The cells were treated with tanshinone IIA at the indicated concentrations followed by 10 mM glutamate exposure. The apoptotic cells were determined using Annexin V/PI staining and flow cytometric analysis (a–e). The apoptosis rate was calculated as the percentage of Annexin V-positive cells in total cells, and the data are presented as mean ± SEM of three independent experiments (f). Tan IIA: tanshinone IIA; Glu: glutamate. ^∗^*p* < 0.05 compared to the cells exposed to glutamate alone.

Previous studies have shown that excessive glutamate can trigger apoptotic changes, such as nuclear shrinkage and chromatin condensation, in neuronal cells [34]. As shown above, tanshinone IIA was able to prevent the loss of MMP induced by glutamate in SH-SY5Y cells. Since MMP loss is an early and key event during apoptosis [35], we tested whether tanshinone IIA could inhibit glutamate-induced cell apoptosis using Annexin V/PI staining and flow cytometry. As shown in Figure 3, the apoptosis rate of glutamate-exposed SH-SY5Y cells was increased to 25.7% as compared to that of the control cells (5.6%), demonstrating the cytotoxic effect of glutamate. When the glutamate-exposed cells were pretreated with tanshinone IIA at 2.5, 5.0, and 10.0 *μ*M, the apoptosis rates were reduced to 18.6%, 14.3%, and 11.9%, respectively (Figure 3). These results indicate that tanshinone IIA is capable of inhibiting glutamate-induced apoptosis in SH-SY5Y cells.

### 3.6. Tanshinone IIA Regulates Expression of Apoptosis-Related Proteins in Glutamate-Exposed SH-SY5Y Cells

**Figure 4:**
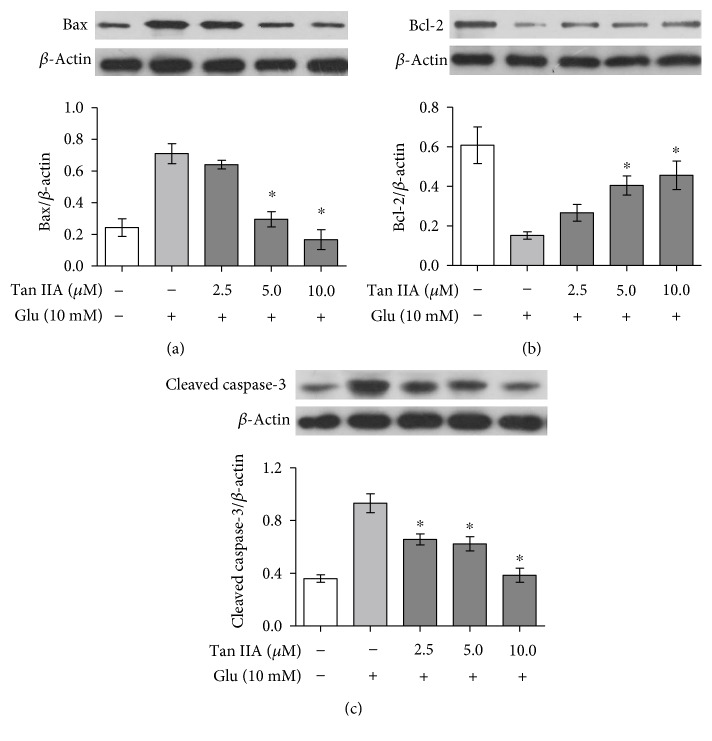
Effect of tanshinone IIA on the protein levels of Bax, Bcl-2, and cleaved caspase-3 in glutamate-exposed SH-SY5Y cells. The cells were treated with tanshinone IIA at the indicated concentrations and then exposed to 10 mM glutamate. Western blot analysis was performed using antibodies against Bax (a), Bcl-2 (b), and cleaved caspase-3 (c), and *β*-actin was used as a loading control. Relative protein levels were quantified by densitometry and normalized to *β*-actin. The data are presented as mean ± SEM of three independent experiments. Tan IIA: tanshinone IIA; Glu: glutamate. ^∗^*p* < 0.05 compared to the cells exposed to glutamate alone.

It is known that Bcl-2 family proteins and caspase family proteases play critical roles in a mitochondria-dependent apoptotic pathway. For instance, Bax, a proapoptotic member of the Bcl-2 family, promotes the loss of MMP and the release of cytochrome c to cytosol, leading to subsequent cleavage and activation of apoptotic executor caspase-3 [36]. However, the antiapoptotic protein Bcl-2 can inhibit Bax expression and suppress caspase-3 activation [36]. Therefore, we investigated whether these apoptosis-related proteins were involved in the antiapoptotic effect of tanshinone IIA using Western blot analysis. As shown in Figure 4, after exposure to 10 mM glutamate for 24 h, the protein levels of Bax and cleaved caspase-3 were increased while that of Bcl-2 was decreased in SH-SY5Y cells, but these trends were reduced by pretreatment with 2.5–10.0 *μ*M tanshinone IIA, suggesting that tanshinone IIA is able to inhibit glutamate-mediated apoptosis through regulation of Bcl-2 family protein expressions and caspase-3 activation.

### 3.7. Tanshinone IIA Inhibits Glutamate-Induced Activation of JNK and p38 MAPK in SH-SY5Y Cells

**Figure 5:**
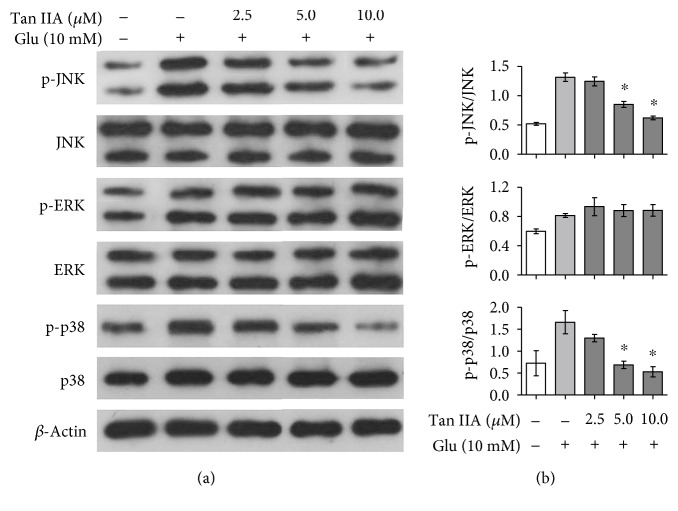
Effect of tanshinone IIA on phosphorylation levels of JNK, ERK, and p38 MAPK in glutamate-exposed SH-SY5Y cells. (a) The cells were treated with tanshinone IIA at the indicated concentrations and then exposed to 10 mM glutamate. Western blot analysis was performed using the indicated antibodies with *β*-actin as a loading control. (b) Quantification of immunoblots was performed by densitometry analysis, and relative phosphorylation levels were expressed as the ratios of phospho-MAPK to total MAPK, respectively. The data are presented as mean ± SEM of three independent experiments. Tan IIA: tanshinone IIA; Glu: glutamate. ^∗^*p* < 0.05 compared to the cells exposed to glutamate alone.

Previous reports have shown that glutamate can induce neuronal apoptosis through activation of MAPK [13], a family of kinases associated with a number of stress signaling pathways. Thus, we investigated the effect of tanshinone IIA on the activation of MAPK, including JNK, ERK, and p38 MAPK, in glutamate-exposed SH-SY5Y cells by Western blot analysis using antibodies able to recognize either total MAPK or their activated, phosphorylated forms. As shown in Figure 5, the protein levels of total JNK1/2, ERK1/2, and p38 MAPK were almost not changed after either 24 h of exposure to 10 mM glutamate or 24 h of pretreatment with 2.5–10.0 *μ*M tanshinone IIA followed by 24 h of 10 mM glutamate exposure. However, the protein levels of the phosphorylated MAPK forms, that is, p-JNK1/2, p-ERK1/2, and p-p38 MAPK, were increased after glutamate exposure, demonstrating the activation of MAPK by glutamate. Interestingly, when the cells were pretreated with tanshinone IIA, the elevated phosphorylation levels of JNK1/2 and p38 MAPK were reduced while that of ERK1/2 remained unchanged as compared to that of the cells exposed to glutamate alone, suggesting the involvement of JNK and p38 MAPK in the protective effect of tanshinone IIA against glutamate toxicity.

## 4. Discussion

An aberrant glutamatergic neurotransmitter system has been shown to be implicated in the pathological process of many neurological diseases [2, 4–8]. In this study, we show that the viability and proliferation of SH-SY5Y cells were markedly decreased while LDH release and cell apoptosis were increased upon treatment with 10 mM glutamate, demonstrating the toxicity inflicted by glutamate in the neuroblastoma cells. Interestingly, pretreatment with tanshinone IIA was able to improve cell viability and proliferation and inhibit LDH release and cell apoptosis in the glutamate-exposed cells (Figures 1, 3, and 1S). Tanshinone IIA has also been previously shown to inhibit 4-aminopyridine-mediated glutamate overrelease in rat cerebrocortical nerve terminals [19]. Together, these findings demonstrate that tanshinone IIA is capable of reducing glutamate-associated neurotoxicity and thus may have a therapeutic potential in excitotoxicity-related disorders.

Excessive excitatory amino acids can stimulate generation of intracellular free radicals and induce oxidative stress, which poses a threat to cell survival [8]. In particular, neurons are more vulnerable to oxidative insults due to their high metabolic activity and oxygen consumption [10]. For example, rats injected with glutamate are shown to develop significant pathological alterations in the brain, including increased ROS level, oxidized lipids, and mitochondrial impairment, and induce neurological behavior deficits [7, 37, 38]. Therefore, reducing oxidative stress is likely a promising strategy to combat excitotoxicity. Here, we show that tanshinone IIA was able to reduce ROS level and MDA and protein carbonyl contents and also enhance activities and expressions of the antioxidant enzymes SOD and CAT in glutamate-exposed SH-SY5Y cells (Table 1), demonstrating its antioxidant capacity. Intriguingly, a number of antioxidant enzymes are known to have distinct isoforms, for example, cytoplasmic Cu/ZnSOD, mitochondrial MnSOD, and extracellular Cu/ZnSOD are the three SOD isoforms in mammalian cells, and it has been demonstrated that excessive excitatory amino acids can reduce the activities and expressions of SOD isoforms [39, 40]. On the other hand, decreased SOD activity and expression are shown to increase excitotoxicity in experimental models [41, 42], while increased enzymatic activity and protein level of SOD isoforms are reported to alleviate excitotoxicity [42, 43]. Interestingly, tanshinone IIA has been shown to modulate SOD isoforms under various conditions, for example, tanshinone IIA can inhibit the production of oxidized low-density lipoprotein through increasing Cu/ZnSOD activity and level in rats with atherosclerotic calcification [44]. Therefore, it is likely that tanshinone IIA has a regulatory effect on SOD isoforms in glutamate-exposed SH-SY5Y cells.

In addition to promoting mitochondrial ROS generation, excessive excitatory amino acids may also disrupt mitochondrial function, including mitochondrial permeability transition, protein carbonylation, and respiratory chain impairment, and cause energy deficiency and release of proapoptotic factors into the cytosol, leading to activation of a caspase-dependent apoptotic pathway [8, 9, 45, 46]. Therefore, maintaining mitochondrial function represents a promising approach to alleviate glutamate neurotoxicity. In our study, tanshinone IIA was found to rescue the loss of MMP and the reduction of ATP content and to inhibit the accumulation of carbonylated mitochondrial proteins in glutamate-exposed cells (Figure 2), demonstrating its capability to maintain mitochondrial integrity and function. Since tanshinone IIA was also capable of attenuating glutamate-mediated ROS overproduction and cell apoptosis (Table 1 and Figure 3), it is likely that its alleviation of glutamate toxicity was through maintenance of mitochondrial function and induction of antioxidant defense. Interestingly, tanshinone IIA has been recently found to inhibit the reduction of ATP content in hypoxia-exposed H9c2 embryonic rat heart-derived cells [47]. Therefore, tanshinone IIA appears to help maintain and restore mitochondrial function under both oxidative and hypoxic stresses.

Increasing evidence has revealed that members of the Bcl-2 and caspase families are involved in glutamate-mediated apoptotic cell death, and regulation of their expression is beneficial to the suppression of glutamate excitotoxicity [48–50]. For example, overexpression of the antiapoptotic Bcl-2 is able to attenuate glutamate-induced neuronal death through reducing mitochondrial superoxide in rat primary hippocampal cultures [51], while inhibition of the proapoptotic Bax and caspase can alleviate glutamate toxicity in a cultured rat's cerebellar granule neurons and retina [52, 53]. Here, we demonstrate that glutamate exposure increased Bax level, decreased Bcl-2 level, and promoted caspase-3 activation in SH-SY5Y cells and also found that tanshinone IIA was able to suppress these changes (Figure 4). This is in agreement with recent studies showing the inhibitory effect of tanshinone IIA on the cytotoxicity of several other neurotoxins, including the Parkinsonism-inducing 1-methyl-4-phenyl-1,2,3,6-tetrahydropyridine and 6-hydroxydopamine, through regulation of apoptosis-related protein expression [17, 54]. Together, these findings support the involvement of apoptosis pathways in the neuroprotective effects of tanshinone IIA. Interestingly, however, tanshinone IIA is also found to induce apoptosis of tumor cells by upregulating expression of proapoptotic proteins [55]. These seemingly conflicting observations suggest that tanshinone IIA may initiate context-dependent signals and responses in a specific cellular environment; in the current circumstance, for instance, the metabolically stressed neuronal cells and the metabolically modified tumor cells have distinct signaling response modes and gene expression patterns [56]. A similar example is quercetin, a plant flavonoid that reduces hydrogen peroxide-induced apoptosis in human neuroblastoma SK-N-MC cells through downregulation of hypoxia-inducible factor 1 (HIF-1) but also inhibits proliferation of human hepatoma HepG2 cells through induction of HIF-1 [57].

MAPK, a highly conserved family of protein serine/threonine kinases known to be activated by a variety of stresses, are involved in the progression of many diseases including excitotoxicity-related neurodegenerative disorders. For example, glutamate-induced activation of p38 MAPK promotes neuronal degeneration and death, while the p38-specific inhibitor SB203580 prevents glutamate neurotoxicity in neonatal rats [14, 58]. In this study, we found that tanshinone IIA was able to suppress the glutamate-elevated phosphorylation levels of JNK1/2 and p38 MAPK (Figure 5). Since the activation of JNK and p38 MAPK is known to induce apoptosis [59], it is likely that regulation of these MAPK pathways contributes, at least in part, to the antiapoptotic capability of tanshinone IIA. Furthermore, MAPK pathways are recently shown to play a role in neurotransmitter exocytosis, for example, involvement of JNK in the regulation of *N*-methyl-*D*-aspartic acid-evoked glutamate release in mice [60], and therefore, the protective effect of tanshinone IIA against glutamate release [19] may also be associated with the MAPK pathways. However, further investigation is needed to unravel the signaling mechanisms of tanshinone IIA against glutamate neurotoxicity as other pathways may also be involved in the neuroprotective effect of tanshinone IIA. For example, tanshinone IIA is shown to protect PC12 cells against amyloid *β*-protein-induced apoptosis via the PI3K/Akt signaling pathway, an important survival mechanism which is also implicated in glutamate excitotoxicity [12, 61].

## 5. Conclusions

**Figure 6:**
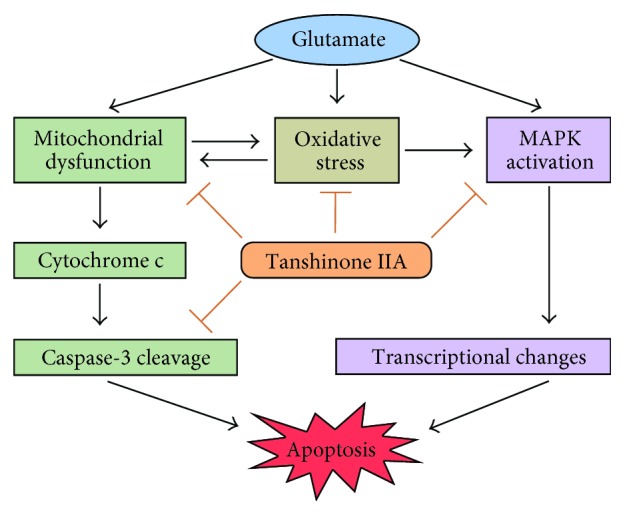
Proposed mechanisms of action of tanshinone IIA against glutamate neurotoxicity. Excessive glutamate exposure induces a series of cytotoxic events including mitochondrial dysfunction and oxidative stress, leading to neuronal apoptosis. However, tanshinone IIA can promote neuronal survival and inhibit cell apoptosis through prevention of mitochondrial dysfunction, modulation of oxidative stress, and suppression of MAPK activation.

In this study, we demonstrate that tanshinone IIA is capable of protecting SH-SY5Y cells against glutamate-induced cytotoxicity. We show that tanshinone IIA not only reduces ROS level and inhibits lipid and protein peroxidation but also increases activities and protein levels of the antioxidant enzymes SOD and CAT under glutamate challenge. Then, we reveal that tanshinone IIA is able to alleviate mitochondrial dysfunction as assessed by membrane potential, mitochondrial protein carbonyl content, and ATP content. We also demonstrate that tanshinone IIA can inhibit cell apoptosis by regulating apoptosis-related proteins, including Bcl-2, Bax, and cleaved caspase-3, in glutamate-exposed cells. We further show that tanshinone IIA suppresses glutamate-induced activation of JNK1/2 and p38 MAPK and thus demonstrate the participation of MAPK pathways in its neuroprotective effect. Collectively, these results support the involvement of multiple signaling pathways in the inhibition of glutamate toxicity by tanshinone IIA (Figure 6) and provide a basis for further studies of its mechanism of action in ameliorating excitotoxicity-related disorders.

## Supplementary Material

FIGURE 1S: Effect of tanshinone IIA on cell viability and proliferation in SH-SY5Y cells under glutamate intoxication. (a) Trypan Blue exclusion rate of the SH-SY5Y cells pretreated with tanshinone IIA at the indicated concentrations for 24 h and then exposed to 10 mM glutamate for another 24 h. (b) BrdU incorporation rate of the SH-SY5Y cells treated as in (a). All data are normalized to the cells without tanshinone IIA treatment and glutamate exposure and presented as mean ± SEM of three independent experiments. Tan IIA, tanshinone IIA. Glu, glutamate. ∗ p < 0.05, compared to the cells exposed to glutamate alone.

## Figures and Tables

**Table 1:** Effect of tanshinone IIA on ROS level, malondialdehyde and protein carbonyl contents, and antioxidant enzymes in glutamate-exposed SH-SY5Y cells.

Treatment^a^		ROS level^b^	MDA content^c^	Protein carbonyl content^d^	Antioxidant enzyme activity^e^		Antioxidant enzyme level^f^	
Tan IIA (*μ*M)	Glu (mM)				SOD	CAT	SOD	CAT
0	0	52.31 ± 2.17	0.89 ± 0.04	11.18 ± 0.25	22.26 ± 0.95	133.61 ± 3.07	1.02 ± 0.02	0.39 ± 0.03
0	10	91.35 ± 6.41^g^	1.21 ± 0.03^g^	38.33 ± 0.49^g^	14.22 ± 0.72^g^	98.18 ± 3.91^g^	0.71 ± 0.03^g^	0.29 ± 0.01^g^
2.5	10	69.12 ± 3.16^h^	0.99 ± 0.05	33.69 ± 0.83^h^	19.54 ± 0.61^h^	113.73 ± 4.39	0.78 ± 0.02	0.30 ± 0.05
5.0	10	63.98 ± 4.07^h^	0.84 ± 0.07^h^	32.78 ± 0.61^h^	20.21 ± 1.36^h^	124.89 ± 5.44^h^	0.94 ± 0.04^h^	0.32 ± 0.03
10.0	10	58.59 ± 5.39^h^	0.76 ± 0.04^h^	30.36 ± 1.35^h^	20.79 ± 1.05^h^	128.42 ± 7.16^h^	1.19 ± 0.07^h^	0.38 ± 0.02^h^

Tan IIA: tanshinone IIA; Glu: glutamate; ^a^pretreatment with tanshinone IIA followed by glutamate exposure; ^b^ROS level, relative DCF fluorescence intensity; ^c^MDA content, nmol/mg proteins; ^d^Protein carbonyl content, pmol/mg proteins; ^e^SOD and CAT activities, U/mg proteins; ^f^SOD and CAT levels, ng/mg proteins; ^g^*p* < 0.05 compared to the control; ^h^*p* < 0.05 compared to glutamate exposure.
