# Supplementary material for: Tanshinone IIA Inhibits Glutamate-Induced Oxidative Toxicity through Prevention of Mitochondrial Dysfunction and Suppression of MAPK Activation in SH-SY5Y Human Neuroblastoma Cells
Source: Oxid Med Cell Longev. 2017 Jun 11;2017:4517486. doi: 10.1155/2017/4517486 (PMC5485345; doi:10.1155/2017/4517486)
Supplement: Supplementary file 1 — FIGURE 1S: Effect of tanshinone IIA on cell viability and proliferation in SH-SY5Y cells under glutamate intoxication. (a) Trypan Blue exclusion rate of the SH-SY5Y cells pretreated with tanshinone IIA at the indicated concentrations for 24 h and then exposed to 10 mM glutamate for another 24 h. (b) BrdU incorporation rate of the SH-SY5Y cells treated as in (a). All data are normalized to the cells without tanshinone IIA treatment and glutamate exposure and presented as mean ± SEM of three independent experiments. Tan IIA, tanshinone IIA. Glu, glutamate. ∗ p < 0.05, compared to the cells exposed to glutamate alone. [file 4517486.f1.doc]

Suppl. Figure 1S


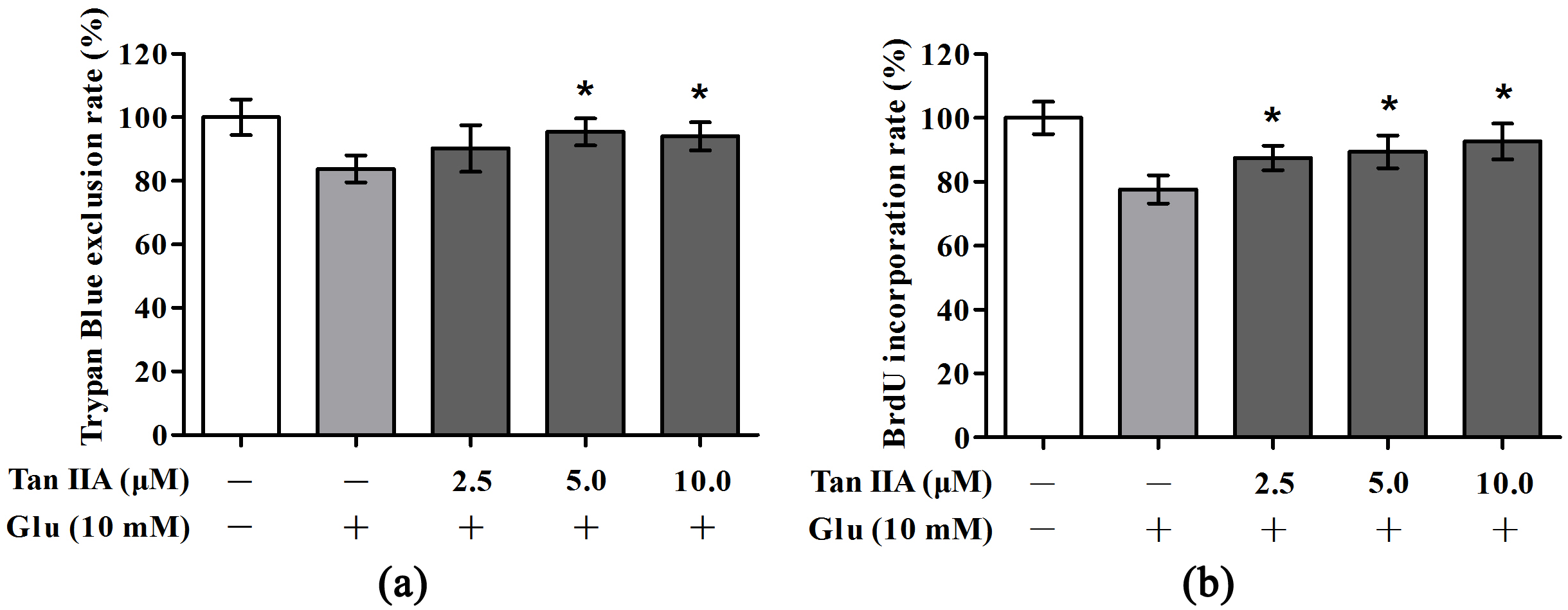


FIGURE 1S: Effect of tanshinone IIA on cell viability and proliferation in SH-SY5Y cells under glutamate intoxication. (a) Trypan Blue exclusion rate of the SH-SY5Y cells pretreated with tanshinone IIA at the indicated concentrations for 24 h and then exposed to 10 mM glutamate for another 24 h. (b) BrdU incorporation rate of the SH-SY5Y cells treated as in (a). All data are normalized to the cells without tanshinone IIA treatment and glutamate exposure and presented as mean ± SEM of three independent experiments. Tan IIA: tanshinone IIA; Glu: glutamate. * *p* < 0.05 compared to the cells exposed to glutamate alone.
